# Assessment of the Chemetrics analyser

**DOI:** 10.1155/S1463924678000115

**Published:** 1978

**Authors:** Robyn White, N. Potezny, T. D. Geary, D. Elston, B. Fuller

**Affiliations:** Institute of Medical and Veterinary Science Box 14 Rundle Street P. 0. Adelaide South Australia 5000 Australia


### Assessment of the Chemetrics analyser

**Robyn White, N. Potezny, T. D. Geary, D. Elston and B. Fuller**

*Institute of Medical and Veterinary Science, Box 14, Rundle Street P.O., Adelaide, South Australia 5000*

### Introduction

The Chemetrics computer controlled chemistry analyser was assessed for its suitability as a general laboratory instrument at this laboratory on behalf of the Committee for Evaluation of Kits and Instrumentation of the Australian Association of Clinical Biochemists. The Chemetrics is manufactured by Chemetrics Corp., La Jolla, California, USA.

To provide additional information on the system, four of the kits recommended for the Chemetrics by the Australian distributor, Calbiochem-Behring Aust. Pty, Ltd., were used to produce whole system performance data. Only those sections of the kit evaluation having a direct bearing on the total system performance are reported here.

The procedure for assessment was based upon the work of Broughton [1], Westgard [2], Barnett [3] and Logan [4], and modified in the light of our own experience.

A detailed technical evaluation is included as part of the evaluation procedure. Figure 1 shows a schematic diagram of the instrument.

### Principle of operation

The Chemetrics analyser is a semi-automatic system incorporating a printer, a sample processor, a UV/vis spectrophotometer with a thermostatically controlled flow cell. A pressure/vacuum system provides pneumatic pressure for activating the sample processor and vacuum for the aspirator.

The samples and reagent mixture were manually pipetted into the reaction vessel. Since this evaluation the company have released a diluter to be used with the system. After the computer is programmed for the test required, the action of the mixing arm is initiated by the 'start' button. This arm is a pneumatically-driven piston whose movement back and forth causes a rocking motion of the reaction vessel.

Following the mixing process, the aspiration arm draws up the mixture and transfers it to the flow cell of the spectrophotometer, where on temperature equilibration, an absorbance reading is made.

The electrical signal input to the computer is analysed and the data reported on the printer.

### Materials and methods

The methods for glucose, urea, alkaline phosphatase and cholesterol were chosen for the evaluation of the Chemetrics as they utilise all the functions of the machine. They were compared with methods routinely used in this laboratory. The analytes used for the test and comparative methods and their principles are summarised below:—

| | *Calbiochem* | *Comparative* |
|---|---|---|
| Glucose | hexokinase | SMAC* (glucose oxidase) |
| Urea | urease | SMAC (diacetyl monoxime) |
| Alkaline Phosphatase | p-nitrophenol | Beckman TR (Calbiochem reagent) |
| Cholesterol | enzymatic | ABA 100 (Abbott Agent) |

*Trade Mark Technicon Equipment Pty. Ltd.

### Precision

1. *Interbatch*

Control materials containing the analytes in low, intermediate and high levels were analysed each day for a period of twenty days.

2. *Intrabatch*

a) A pooled serum was assayed twenty times in one run.
b) Patient sera were assayed in duplicate

### Accuracy

Accuracy was assessed by using material from the College of American Pathologists Comprehensive Chemistry Programme for which consensus values are available and material with glucose levels assigned by the reference hexokinase method.

A number of commercial controls were assayed as a guide to accuracy.

Eighty patients were analysed at the rate of five each day by the test and comparative methods.

### Linearity

Linearity of response was listed for glucose and urea over the concentration range (glucose to 28 mmol/l and urea to 35 mmol/l) by means of aqueous standards. This experiment was repeated eight times.

### Interference

The effect of grossly lipaemic, haemolysed and icteric sera was determined.

### Results

### Precision

The results of the imprecision studies are tabulated in Tables 1, 2, 3 and 4.

Applying Tonk's criteria, that is, a method is acceptable when twice the coefficient of variation is within the allowable limits of error, i.e. 2 CV % < ALE the Calbiochem glucose, urea, alkaline phosphatase and cholesterol methods were acceptable over the range of values analysed.

### Accuracy

We consider bias < ALE from a carefully verified reference serum value indicates that the test method gave a satisfactory measurement of the 'true value'.

Results obtained for glucose on material with assigned values by the reference hexokinase method, College of American Pathologists and commercial control sera were very close to the stated values. This was also true for urea values obtained for the College of American Pathologists and commercial control sera.

For alkaline phospatase the value for the Technicon SMA 12/60 stated in the information provided on the insert of each control serum was selected. This methodology is widely used

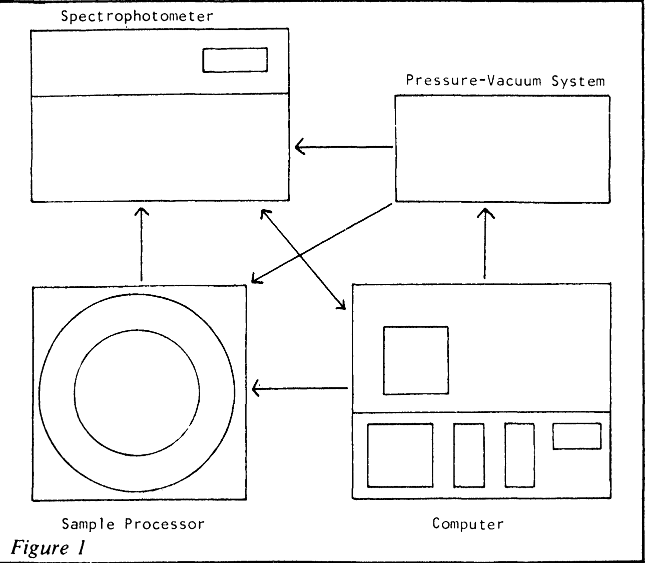

*Figure 1*

**Table 1 Glucose imprecision**

| Interbatch | Mean (mmol/l) | SD | 2 CV % | ALE |
|---|---|---|---|---|
| Low | 2.38 | 0.079 | 6.6 | 10.0 |
| Medium | 4.89 | 0.104 | 4.3 | 10.0 |
| High | 11.80 | 0.271 | 4.6 | 10.0 |
| Intrabatch | | | | |
| a) Pooled serum | 6.34 | 0.086 | 2.70 | |
| b) Patient samples | | | | |
| — difference between duplicates | 0.153 | 0.146 | | |

**Table 2 Urea imprecision**

| Interbatch | Mean (mmol/l) | SD | 2 CV % | ALE |
|---|---|---|---|---|
| Low | 3.27 | 0.161 | 9.8 | 12.0 |
| Medium | 7.20 | 0.206 | 5.7 | 12.0 |
| High | 18.40 | 0.499 | 5.5 | 12.0 |
| Intrabatch | | | | |
| a) Pooled serum | 8.00 | 0.130 | 3.26 | |
| b) Patient samples | | | | |
| — difference between duplicates | 0.175 | 0.163 | | |

**Table 3 Alkaline phosphatase imprecision**

| Interbatch | Mean (U/l) | SD | 2 CV % | ALE |
|---|---|---|---|---|
| Low | 19.2 | 1.76 | 18.3 | 20.0 |
| Medium | 82.8 | 3.21 | 7.7 | 20.0 |
| High | 353.6 | 19.68 | 11.1 | 20.0 |
| Intrabatch | | | | |
| a) Pooled serum | 80.9 | 1.09 | 1.35 | |
| b) Patient samples | | | | |
| — difference between duplicates | 1.38 | 1.39 | | |

**Table 4 Cholesterol imprecision**

| Interbatch | Mean (mmol/l) | SD | 2 CV % | ALE |
|---|---|---|---|---|
| Serachol | 9.38 | 0.168 | 2.6 | 10.0 |
| Lipidtrol | 7.79 | 0.217 | 5.6 | 10.0 |
| Intrabatch | | | | |
| a) Pooled serum | 5.07 | 0.057 | 2.24 | |
| b) Patient samples | | | | |
| — difference between duplicates | 0.09 | 0.116 | | |

and has a known bias. The differences between the stated and observed values for alkaline phosphatase and cholesterol were small considering the differences in methodologies used.

When the Student's "t" test was applied to patient comparison results, it showed there was a significant difference between the test and comparative methods. However, a statistically significant result may not be significant clinically. By applying further criteria, a kit is unacceptable if:—

1. The bias exceeds the allowable limits of error.
2. The number of false clinical decisions exceeds 5%.

Based upon these criteria, the differences between the test and comparative methods for each of the patient comparisons were not clinically significant.

### Linearity

The glucose method was linear to 22.0 mmol/l, becoming approximately 4% lower at 28.0 mmol/l.

The urea method was linear to 35.0 mmol/l.

### Interference

Abnormally coloured serum interfaced with the glucose method. Running a blank compensated for the difference in most cases. There were occasions, however, when the blank did not adequately compensate for colour interference. This apparent anomaly needs further investigation.

### Technical assessment

The system is well constructed and finished in baked enamel to provide a durable finish.

The interconnecting cables are short which tends to restrict the positioning of the modules. The layout of the electrical/electronic hardware gives good service accessibility with the printed circuit board umbilical cords long enough to provide for the use of extender boards.

The Chemetrics system can be used with other spectrophotometers provided they are fitted with a Chemetrics interface and flow cell. The compressor is sturdy and silent with anti-vibration support feet.

The spectrophotometer was tested for wavelength accuracy, linearity, stray light, drift, carryover, flow cell temperature and thermal response. Results showed that the wavelength was accurate to within a nanometre, linearity extended to at least 1.75A and no problems with stray light, carry-over or drift were encountered. The temperature of the flow cell as indicated by the Chemetrics computer was not the true temperature and, aspirating a volume of 0.8 ml the thermal response of the flow cell was well outside the ±0.2°C regulation claimed by the manufacturer.

Sample evaporation from the reaction vessel was negligible over a thirty minute period at a room temperature of 25°C.

To test the mixing action, 30 test runs of 5 $\mu$l stock green solution and 1 ml of distilled water were mixed and read at 400 nm. The coefficient of variation was 0.015%, indicating complete mixing of sample.

### Discussion

Several days were allowed, before the evaluation was commenced, during which detailed instruction in the operation of the instrument was given.

Other than the incorrect indicated temperature of the flow cell, the slow thermal response time and what appeared to be inadequate blanking of highly lipaemic sera when testing for glucose, the instrument performed very reliably during the evaluation period. There are problems which require further investigative work.

Over the two months of the evaluation the simple cleaning procedure recommended by the manufacturer for the flow cell proved adequate and no problem with drift or carry-over was encountered.

The data generated on the patient samples by the comparative methods were within the acceptable limits of clinical criteria.

The methods for glucose and urea depend on a one point calibration. This assumes that the method is linear for a wide range of values. This proved to be the case for both analytes with glucose linear to 22 mmol/l with only a slight decrease in linearity to 28 mmol/l and urea linear to 35 mmol/l.

The precision and accuracy of the four methods tested were considered acceptable. When the inter and intra-batch imprecision data are considered it is apparent that the total system achieves a high degree of precision.

Although the four kits reported on were acceptable by all criteria, the calcium kit recommended proved unacceptable. This emphasises the necessity to fully evaluate every candidate method.

The small sample size required and the computation facilities available with the system should make it suitable for many laboratories.

In conclusion, the kits have been used as a means to evaluate the performance of this analyser. In doing so, it is important to realise that the total variance of the system has as components the errors due to the kits as well as the Chemetrics analyser.

The total system variance was well within the chosen criteria of acceptability. Therefore the performance of the analyser itself must have been acceptable.

REFERENCES
[1] Broughton, P. M. G., Gowenlock, A. H., McCormack, J. J., and Neill, D. W., (1974). A revised Scheme for the Evaluation of Automotive Instruments for use in Clinical Chemistry. *Am. Clin. Biochem,* **11,** 207.
[2] Westgard, S. O., Carey, R. N., Wold, S. (1974). Criteria for Judging Precision and Accuracy in Method Development and Evaluation. *Clin. Chem.* **20,** 825-833.
[3] Barnet, R. N. and Jouden, W. J. (1970). A revised scheme for the comparison of quantitative methods. *American Journal of Clinical Pathology,* **54,** 454-462.
[4] Logan, J. E. (1972). Evaluation of commercial kits. *C.R.C. Critical Reviews in Clinical Laboratory Sciences,* **3,** 271-289.

### Short Communication

### A simple inert pump for use with concentrated acids in automatic systems

**R. G. Lidzey**

*Automatic Methods and Computing Group, Laboratory of the Government Chemist, Cornwall House, Stamford St., London, SE1 9NQ, UK.*

**SAMPLE** pretreatment is an important part of automatic analysis and the transfer of liquids such as corrosive acids and organic solvents is frequently required. Commercially available pumps and dispensers are often not completely inert, interfering substances can be leached from components into the analytical stream and thereby affect subsequent analytical measurements. When inert pumps are available they are rarely suitable for laboratory scale automated analytical systems, either because their flow rates are far too high, or because they are very expensive. Considerable efforts have been made in this Laboratory to develop inert pumping and dispensing assemblies, particularly for use with hot concentrated sulphuric and nitric acids.

A simple pump has been used on a previous occasion in this Laboratory [1, 2] for the transfer of acid digests in trace metal analysis. A rather more flexible system is being used now for adding nitric acid to a distillation flask at a few millilitres per minute. It consists of an assembly of mainly commercial fittings and may be described as a pump or dispenser. One item which was constructed, a non-return valve shown in figure 1, was made from PTFE and Kel-F and has screwed end connections suitable for Chromatronix or Altex flanged PTFE tube couplings. Figure 2 is a diagram of the assembly and shows the arrangement used, liquid is drawn in via one non-return valve when the syringe is operated by an air cylinder which is controlled by a 5 port valve. Settings on this valve limit the rate of filling or emptying of the syringe. A timer determines the

*Figure 1.*

